# Isolation and characterization of novel *Staphylococcus aureus* bacteriophage Hesat from dairy origin

**DOI:** 10.1007/s00253-024-13129-y

**Published:** 2024-04-15

**Authors:** Barbara Turchi, Claudia Campobasso, Arianna Nardinocchi, Jeroen Wagemans, Beatrice Torracca, Cédric Lood, Graziano Di Giuseppe, Paola Nieri, Fabrizio Bertelloni, Luca Turini, Valeria Ruffo, Rob Lavigne, Mariagrazia Di Luca

**Affiliations:** 1 Department of Veterinary Sciences, University of Pisa, Viale Delle Piagge 2, 56124 Pisa, Italy; 2 Department of Biology, University of Pisa, Via San Zeno 37, 56127 Pisa, Italy; 3 Department of Biosystems, KU Leuven, Kasteelpark Arenberg 21, Box 2462, 3001 Louvain, Belgium; 4 Department of Microbial and Molecular Systems, Centre for Microbial and Plant Genetics, KU Leuven, Kasteelpark Arenberg 20, Box 2460, 3001 Leuven, Belgium; 5 Department of Pharmacy, University of Pisa, Via Bonanno Pisano 6, 56126 Pisa, Italy

**Keywords:** Bacteriophage, *Staphylococcus aureus*, Milk, Temperate phage, Prophage

## Abstract

**Abstract:**

A novel temperate phage, named Hesat, was isolated by the incubation of a dairy strain of *Staphylococcus* *aureus* belonging to spa-type t127 with either bovine or ovine milk. Hesat represents a new species of temperate phage within the *Phietavirus* genus of the *Azeredovirinae* subfamily. Its genome has a length of 43,129 bp and a GC content of 35.11% and contains 75 predicted ORFs, some of which linked to virulence. This includes (i) a pathogenicity island (SaPln2), homologous to the type II toxin-antitoxin system PemK/MazF family toxin; (ii) a DUF3113 protein (*gp30*) that is putatively involved in the derepression of the global repressor Stl; and (iii) a cluster coding for a PVL. Genomic analysis of the host strain indicates Hesat is a resident prophage. Interestingly, its induction was obtained by exposing the bacterium to milk, while the conventional mitomycin C–based approach failed. The host range of phage Hesat appears to be broad, as it was able to lyse 24 out of 30 tested *S. aureus* isolates. Furthermore, when tested at high titer (10^8^ PFU/ml), Hesat phage was also able to lyse a *Staphylococcus muscae* isolate, a coagulase-negative staphylococcal strain.

**Key points:**

*• A new phage species was isolated from a Staphylococcus aureus bovine strain.*

*• Pathogenicity island and PVL genes are encoded within phage genome.*

*• The phage is active against most of S. aureus strains from both animal and human origins.*

**Supplementary Information:**

The online version contains supplementary material available at 10.1007/s00253-024-13129-y.

## Introduction

Antimicrobial resistance (AMR) has emerged as one of the leading public health threats of the twenty-first century. Recent estimates attribute 1.27 million deaths to bacterial AMR in 2019. Among these, *Staphylococcus aureus* ranks second on the list of microorganisms responsible for the fatal AMR burden occurring in high-income countries (Murray et al. 2022). Indeed*, S. aureus* is responsible for a plethora of infections that range from nosocomial infections that are associated with high morbidity and mortality rates in humans to infection in food animals, such as cows and sheep (Morgan 2008).

In particular, *S. aureus,* a commensal bacterium of the skin and mucosa in warm-blooded animals, is able to survive in the environment and be transmitted via the air, contacts between individuals, secretions, etc., in both humans and ruminants (Kozajda et al. 2019). Although various studies have shown that nasal carriage is an important reservoir, *S. aureus* is still considered an important mastitis pathogen in ruminants (Krishnamoorthy et al. 2021). Therefore, *S. aureus* is a dangerous One Health concern for animal and human health (McCarthy et al. 2012).

*S. aureus* is a highly clonal species characterized by a core genome whose diversification is driven by homologous recombination at the species level or point mutation within closely related strains (Feil et al. 2003; Everitt et al. 2014). As for homologous recombination, biodiversity of *S. aureus* strains primarily comes from mobile genetic elements, including pathogenicity islands, plasmids, transposons, staphylococcal cassette chromosomes (SCC), and prophages (Everitt et al. 2014). All *S. aureus* genomes sequenced to date contain one to four prophages (Lindsay 2010). Bacteriophages provide their host fitness and different virulence factors such as Panton-Valentine leukocidin (lukSF), exfoliative toxin A (eta) (Yamaguchi et al. 2001), the cell wall–anchored protein SasX (Li et al. 2012), and the immune evasion cluster (IEC) composed of enterotoxin S (sea), staphylokinase (sak), the chemotaxis inhibitory protein (chp), and the staphylococcal complement inhibitor (scn) (van Wamel et al. 2006). Although some phages can readily transduce between staphylococcal species, horizontal gene transfer is generally limited within the species and clonal complexes with the same restriction-modification (R-M) system (Waldron and Lindsay 2006).

The increasing number of resistant *S. aureus* strains, especially methicillin-resistant *S. aureus* (MRSA), and the huge impact on animal health and welfare causing major economic losses in livestock production drive the research for alternative or complementary approaches to fight them (Kasela et al. 2023). Currently, one of the most promising strategies being explored is phage therapy which is based on the application of bacteriophages as antimicrobials (Strathdee et al. 2023). For phage therapy, strictly virulent phages are suggested to be used, while natural temperate phages are not considered suitable, due to their ability to integrate in the host genome and transfer virulence and resistance genes (Gordillo Altamirano and Barr 2019). However, advances in genome sequencing and synthetic biology offer opportunities to explore temperate phages or their derivatives not only in therapy, with the creation of lytic and tailored variants, but also in the rapid detection of pathogenic bacteria (Monteiro et al. 2019).

Here, in a first attempt to isolate lytic *S. aureus* phages from milk to be applied in phage therapy, a new species of a temperate phage, named Hesat, was isolated and characterized. Hesat phage was induced from a dairy strain of *S. aureus* incubated with either bovine or ovine raw milk during an enrichment procedure. Both genotyping and phenotypic characterization of the phage were carried out, including genome analysis, as well as phage host range versus a panel of *S. aureus* and coagulase-negative staphylococcal strains from veterinary and human origin. Finally, the potential application of the phage or its components for the detection of *S. aureus* strains has been discussed.

## Material and methods

### Staphylococcal isolates

Seven *S. aureus* isolates (18, 30, 119, 916, 852, 153, 224) from sheep and bovine milk were employed as hosts to isolate new bacteriophages from raw milk (Table 1). They were chosen from a proprietary strain collection of the Department of Veterinary Science (University of Pisa) based on different geographical origins, animal species (sheep or cow), and type of sample (bulk tank milk, individual milk) and submitted to genotypic characterization. Isolates were genotyped by amplifying the *spa* gene and analyzing the sequences obtained according to the protocol by Mellmann et al. (2006) with primers spa-1113f (5′-TAA AGA CGA TCC TTC GGT GAG C-3′) and spa-1514r (5′-CAG CAG TAG TGC CGT TTG CTT-3′). Next, the amplicons were sent to BMR Genomics (Padua, Italy) for Sanger sequencing. For sequencing analysis, software described by Sullivan et al. (2019) (spaTyper) (https://github.com/mjsull/spa_typing) was employed, modified to be used with Python 3. Once a FASTA file was obtained, the software identifies the repeats and their order within the *spa* gene sequence, thus generating a spa type which is then retrieved from a specific database (http://spaserver2.ridom.de/). Isolates used as hosts were further characterized for the presence in their genome of prophages belonging to the serogroups A, B, F, L, and D (Pantůček et al. 2004); for the presence of *mecA* and *mecC* genes (Zhang et al. 2005; Cuny et al. 2011), related to methicillin resistance; and for the presence of enterotoxin genes (*sea*, *seb*, *sec*, *sed*, and *see*) (Normanno et al. 2007). In addition, the susceptibility to methicillin was phenotypically tested by standard antibiograms performed according to European Committee on Antimicrobial Susceptibility Testing (EUCAST) recommendations (EUCAST Disk Diffusion Method for Antimicrobial Susceptibility Testing-Version 10.0, January 2022, www.eucast.org). The following antibiotics were tested: cefoxitin (FOX, 30 mg), ciprofloxacin (CIP, 5 mg), levofloxacin (LEV, 5 mg), gentamycin (CN, 10 mg), tobramycin (TOB, 10 mg), erytromycin (E, 15 mg), tetracycline (TE,30 mg), linezolid (LZD, 30 mg), and sulfamethoxazole-trimethoprim (SXT, 25 mg).

**Table 1:** *S. aureus* isolates included in the study

	Isolate ID	Source	Geographic origin	Spa type	Prophage^a^	Methicillin resistance^b^	Phenotypic resistance	Enterotoxin genes^c^
Dairy strains	18	Bulk tank ovine milk	Viterbo (Italy)	t127	B	MRSA (*mecA)*	FOX, E	n.d
	30	Bulk tank bovine milk	Grosseto (Italy)	t2953	F	MSSA	n.d	*sea*
	119	Bulk tank bovine milk	Grosseto (Italy)	t2953	F	MSSA	n.d	*sea*
	916	Bulk tank bovine milk	Bari (Italy)	t127	B	MRSA (*mecA*)	FOX, TOB, E, TE	n.d
	852	Bulk tank bovine milk	Matera (Italy)	t688	n.d	MRSA (*mecA*)	FOX, E, TE	n.d
	153	Individual mastitic ovine milk	Pistoia (Italy)	t20372	Fb, A	MSSA	TE	n.d
	224	Individual mastitic ovine milk	Pistoia (Italy)	t1166	Fb, A	MSSA	n.d	*sec*
Human strains	VAR	Pyelonephritis	Udine (Italy)	t148	n.a	MRSA (*mecA*)	FOX	n.a
	VT	Osteomyelitis	Verona (Italy)	t127	n.a	MSSA		n.a
	DS	Osteomyelitis	Rovigo (Italy)	t034	n.a	MRSA (*mecA*)	FOX	n.a
	5376	Osteomyelitis	Pisa (Italy)	t032	n.a	MSSA		n.a
	DT	Cardiac implantable electronic device	Napoli (Italy)	t2062	n.a	MRSA (*mecA*)	FOX	n.a
	C2	Prosthetic joint infection	Berlin (Germany)	t379	n.a	MRSA (*mecA*)	FOX	n.a
	C4	Prosthetic joint infection	Berlin (Germany)	t015	n.a	MRSA (*mecA*)	FOX	n.a
	C9	Prosthetic joint infection	Berlin (Germany)	t008	n.a	MRSA (*mecA*)	FOX	n.a
	C10	Prosthetic joint infection	Berlin (Germany)	t002	n.a	MRSA (*mecA*)	FOX	n.a
	C16	Prosthetic joint infection	Berlin (Germany)	t5393	n.a	MSSA		n.a
	190,809–53	Prosthetic joint infection	Leuven (Belgium)	t002	n.a	MSSA		n.a
	190,605–84	Prosthetic joint infection	Leuven (Belgium)	t190	n.a	MSSA		n.a
	V-190823–86	Prosthetic joint infection	Leuven (Belgium)	t084	n.a	MSSA		n.a
	V-190830–88	Prosthetic joint infection	Leuven (Belgium)	t091	n.a	MSSA		n.a
	V-190821–119	Prosthetic joint infection	Leuven (Belgium)	t1589	n.a	MSSA		n.a
	AOMu05	Prosthetic joint infection	Davos (Switzerland)	t192	n.a	MSSA		n.a
	AOMu81	Prosthetic joint infection	Davos (Switzerland)	t003	n.a	MRSA (*mecA*)	FOX	n.a
	AOMu100	Prosthetic joint infection	Davos (Switzerland)	t032	n.a	MRSA (*mecA*)	FOX	n.a
	AOC19	Prosthetic joint infection	Davos (Switzerland)	t1858	n.a	MSSA		n.a
	AOC42	Prosthetic joint infection	Davos (Switzerland)	t062	n.a	MSSA		n.a
	ATCC 43300			t007	n.a	MRSA (*mecA*)	FOX	n.a
	ATCC 25923			t021	n.a	MSSA		n.a
	ATCC 6538			t3297	n.a	MSSA		n.a

^a^Pantůček et al. 2004; ^b^Zhang et al. 2005; ^c^Normanno et al. 2007; *n.d.* not detected, *n.a.* not assayed, *FOX* cefoxitin, *E* erythromycin, *TOB* tobramycin, *TE* tetracycline

The lytic potential of new isolated phages was tested against all dairy *S. aureus* isolates and a further set of 20 human *S*. *aureus* isolates (Table 1). These staphylococcal strains were collected from patients suffering from pyelonephritis, osteomyelitis, cardiac implantable electronic devices, and prosthetic joint infections. The susceptibility to methicillin of human isolates was phenotypically tested by standard antibiograms performed according to European Committee on Antimicrobial Susceptibility Testing (EUCAST) recommendations (EUCAST Disk Diffusion Method for Antimicrobial Susceptibility Testing- Version 10.0, January 2022, www.eucast.org). In addition, the presence of *mecA* and *mecC* gene was also assessed with PCR-based methods previously mentioned. Human *S. aureus* isolates were also subjected to spa typing as previously reported. The collection was supplemented with standard laboratory strains of *S. aureus* (ATCC 43300, ATCC 25923, and ATCC 6538).

Nine additional staphylococcal isolates from bulk tank ovine milk and belonging to the coagulase-negative group were also included in host range tests (*Staphylococcus epidermidis* 46A, *Staphylococcus simulans* 60B, *Staphylococcus chromogenes* 50C, *Staphylococcus equorum* 81A, *Staphylococcus arlettae* 32B, *Staphylococcus xylosus* 69A, *Staphylococcus auricularis* 68C, *Staphylococcus jettensis* 33C, *Staphylococcus muscae* 53C).

All the isolates were stored at − 20 °C in BHI broth (Thermo Fisher Diagnostics, Milan, Italy) with 15% glycerol.

### Dairy sample collection and bacteriophage isolation

To isolate *S. aureus* bacteriophages from a dairy environment, 83 samples were collected from four animal farms (one cattle farm and three sheep farms) and a dairy farm located in Tuscany (Italy). Samples were represented by individual raw bovine milk (*n* = 21); individual raw sheep milk (*n* = 60, 20 samples from each dairy sheep farm); sheep milk whey from the dairy wastewater well (*n* = 1); sheep milk whey from the ricotta cheese–making process (*n* = 1). Raw milk samples were collected from healthy animals. Each sample was tested for the presence of bacteriophages against the seven *S. aureus* isolates (18, 30, 119, 916, 852, 153, 224) from sheep and bovine milk (Table 1) by an enrichment procedure, as previously described (García et al. 2009) with minor modifications. Each isolate was individually inoculated in BHI broth with the addition of 100 μl of different milk samples for the enrichment step, and after incubation (37 °C, 24 h) with shaking, all the samples were centrifuged (11,500 *g* for 5 min) and filtered with syringe filters (0.22 μm pore diameter) to collect phage particles. Then, each filtrate was subjected to a spot test using as host the same isolate employed in the enrichment. The presence of lysis halos indicating the presence of phages was checked.

### Bacteriophage purification, amplification, and storage

For phage purification, the tip of a sterile paper strip was dipped in the phage suspension. The strip was subsequently streaked on a Petri dish containing a bacterial lawn of the isolate used in the enrichment step. The plates were incubated overnight at 37 °C. From each plate, one plaque was picked with a sterile tip. Plaques were passaged several times to obtain pure bacteriophage cultures. A 5 ml aliquot of soft TSA agar was added, and plates were incubated at 37 °C for 18–24 h. Subsequently, 5 ml of saline magnesium (SM) buffer was added, and plates were kept at room temperature for 18 h with shaking. The SM buffer was then recovered, centrifuged at 3200 *g* for 10 min, and filtered with syringe filters (0.22 μm pore diameter). Phage lysates were stored at − 20 °C with the addition of 50% sterile glycerol. For phage amplification, 0.1 ml of phages (approximatively 1 × 10^5^ PFU/ml) was added to 0.1 ml of an overnight *S. aureus* culture (MOI 0.001). Five milliliters of soft TSA agar was added, and the mixture was poured onto TSA plates which were incubated at 37 °C for 18–24 h. Phages were recovered as described above and stored at 4 °C.

### Prophage induction by BHI, raw milk, and mitomycin C

To assess the induction of prophage by raw milk incubation, 100 μl of raw and boiled (15 s at 900 W in a microwave) milk samples from either ovine or bovine was added to exponentially grown bacterial cells in BHI broth (1.4 ml). A control sample without milk addition was also included. After 24-h incubation at 37 °C with shaking, samples were centrifuged at 11,500 *g* for 5 min and filtered and we performed spot testing to assess the presence of released phages. Negative controls, *i.e.*, aliquots of the tested ovine and bovine milk samples, were also included. These samples were treated following the same protocol except for the inoculation of *S. aureus* 916 cells.

Prophage induction was also performed treating cells with mitomycin C (Schaefler et al. 1976). Briefly, 25 ml of *S. aureus* 916 cells exponentially grown (OD_600_ = 0.2) in BHI medium was treated with mitomycin C (1 μg/ml), including an untreated control. The OD of both cultures was monitored at intervals of 30–60 min until the OD of treated cell culture decreased. A volume of 5 ml of each culture was collected, centrifuged (4000 *g* for 10 min), and filtered with 0.22-μm filters. The filtered samples were tested by spot assay on a growing bacterial lawn of *S. aureus* 916, incubating overnight at 37 °C.

### Bacteriophage and bacterial genome sequencing, assembly, and phage annotation

Phage DNA was extracted using the Zymo Research DNA Clean & Concentrator™-5 kit. The phage solution was previously treated with DNAse I and RNAse A to remove bacterial genetic material and with proteinase K to digest the capsid proteins and release phage genomic DNA (Green et al. 2012). Genomic DNA of *S. aureus* strain 916 was isolated using the DNeasy UltraClean microbial kit (Qiagen, Milan, Italy), following the manufacturer’s protocol. Both phage and bacterial genome samples were assessed using the NanoDrop™ Lite Spectrophotometer (Thermo Scientific™, Milan, Italy). Whole genome sequencing was performed at KU Leuven (Laboratory of Gene Technology) in Belgium. Illumina sequencing libraries were created using the Nextera Flex DNA Library Kit. For each sample, raw data were submitted to the BV-BRC online platform v3.6.12 (Wattam et al. 2017) and assembly was performed using Unicycler v0.4.8 (Wick et al. 2017), following default settings. BLASTn (Altschul et al. 1990) was used to screen the NCBI nucleotide collection database (nr/nt) to find similar sequences to the phage genome sequence. Query sequences were realigned to their best match found. The final FASTA sequences were provided to the BV-BRC platform to run genome annotations using RASTtk (Brettin et al. 2015), followed by manual functional annotation comparing BV-BRC predicted CDSs against the non-redundant GenBank protein database (Kelley and Sternberg 2009) using BLASTp (Altschul et al. 2005). The prediction of transmembrane domains was performed using DeepTMHMM (Hallgren et al. 2022). Moreover, the assembled genome was screened for the presence of acquired antibiotic resistance genes by ResFinder v4.1 (Zhang et al. 2005; Camacho et al. 2009; Bortolaia et al. 2020). Easyfig v2.1 (Sullivan et al. 2011) was used for visualization of the genome map. The analysis of intergenomic distance was performed through VirClust (Moraru 2021). The characterization of the endolysins was first performed with HMMER v3.4 (Finn et al. 2011), to identify the putative functional domains within the protein, while their structure was predicted through ColabFold v1.5.5 (Mirdita et al. 2022) and visualized in PyMOL v2.5.4 (Schrödinger, LLC 2015).

The assembly of the bacterial reads was performed using SPAdes v3.15.4 (Prjibelski et al. 2020) and the genome was annotated using Prokka v1.14.6 (Seemann 2014). The assessment of the clonal complex of the target bacterial strain was performed through BIGSdb software, available on PubMLST website (Jolley et al. 2018).

To assess whether the isolated phages were prophages of their bacterial hosts, phage and bacterial genome sequences were aligned to each other. The alignment was performed with BLASTn submitting phage sequence and the bacterial genome sequence as query and subject sequences, respectively, and following default settings. Moreover, the reads obtained from the bacterial genome sequencing were mapped against the phage Hesat genome with BWA-MEM v0.7.17 (Li 2013) and the mapping was visualized with weeSAM (https://github.com/centre-for-virus-research/weeSAM).

Finally, to assess the presence of a specific Hesat DNA sequence within the bacterial strain DNA, a PCR was performed both on the glycerol stock and two samples of the genomic DNA of the *S. aureus* 916 strain. Two phage lysate samples were also added as positive control. The primers were designed starting from the phage Hesat sequence (5′-AGG CGT GAG TTT ACT GTA TAT GG-3′ as forward primer and 5′-GTT GTC ACC TCC CGC TTA CA-3′ as reverse one) to have a PCR product 405-bp long. The PCR samples were prepared as follows: each mix of 30 μl final volume contained 2 μl sample (the bacterial genome samples with a concentration of 15.6 and 24.9 ng/μl, respectively), 3 μl of 10 × DreamTaq Green Buffer (20 mM MgCl_2_ included) (Thermo Fisher Scientific), 0.5 μl dNTP (20 μM) (Eurogentec), 0.5 μl of each primer (20 μM) (IDT), and 0.5 μl 1:10 diluted DreamTaq DNA Polymerase (5U/ml) (Thermo Fisher Scientific).

The PCR was performed through one initial cycle at 95 °C for 8 min; 30 cycles of denaturation at 95 °C for 30 s, annealing at 54 °C for 30 s, and extension at 72 °C for 1 min; and single final extension at 72 °C for 5 min and then held at 10 °C. PCR products were separated by gel electrophoresis on a 2% agarose gel for 30 min at 120 V.

### Identification of prophages similar to Hesat in staphylococcal species

To identify prophage sequences phylogenetically related to Hesat, four relevant genes from its genome (the integrase *gp1*; the pathogenicity island *gp3*; the PVL *gp31*; the major capsid protein *gp47*) were selected and their sequences were submitted to BLASTn, limiting the research to the *Staphylococcus* genus (taxid:1279) and selecting 5000 as maximum number of target sequences. The results obtained from the four analyses were compared to select only those strains sharing all four genes. The genome of the strains so defined were uploaded to PHASTER (Zhou et al. 2011; Arndt et al. 2016), to predict putative prophage regions. Only intact regions were downloaded, and their sequences were aligned to Hesat sequence using BLASTn (Camacho et al. 2009), to assess which prophages belonged to its same genus (similarity threshold of 70%). Finally, the most similar prophages were aligned to Hesat to highlight genomic similarity using VipTree v3.6 (Nishimura et al. 2017).

### Bacteriophage host range

The lytic activity of the selected bacteriophage was determined by a spot test (Clokie and Kropinski 2009). A drop (10 μl) of bacteriophage lysate at high (10^8^ PFU/ml), medium (10^6^ PFU/ml), and low (10^4^ PFU/ml) titer was spotted on the surface of TSA plates, each one prepared with a lawn of a different *S. aureus* isolate (Table 1). The presence of lysis halos indicating the presence of phages was checked.

## Results

### Bacteriophage isolation

Seven *S. aureus* strains among those analyzed were chosen as bacterial hosts based on their heterogeneity in terms of phenotype and genotype characteristics (Table 1). Five different spa types were detected, one of which was not present in the Ridom SpaServer. This spa type was therefore submitted to the database (spa server accession number: 204295). The novel type was assigned to the spa-type t20372. *S. aureus* strain 18, 916, and 852 harbored *mecA* gene, while all the strains were negative for the presence of *mecC*. As for enterotoxins genes, *S.aureus* 30 and 119 possessed *sea* gene coding for enterotoxin A and *S. aureus* 224 *s* gene for enterotoxin C.

Sixty-nine milk samples out of 83 (83%) incubated with *S. aureus* 916 (spa type t127) tested positive for the presence of bacteriophages, while milk incubation with other dairy strains did not allow the detection of phages. The recovery of bacteriophages active against *S. aureus* 916 does not appear to be linked to the type or origin of milk samples, as all the sets of milk samples from different farms resulted in a high percentage of samples positive for phage presence. In particular, when ovine milk collected from the three different farms was used, phage particles were obtained in 100%, 85%, and 75% samples, while using cow milk, phages were obtained from 71% of samples. At the same time, when *S. aureus* 916 was inoculated in boiled milk, no phage particles were obtained. Raw milk samples were also incubated in BHI broth without bacteria to check for the presence of phages. However, when the spot test was performed, none of them showed clear plaques. In addition, 1 μg/ml mitomycin C was not able to induce any phage from *S. aureus* strain 916. Ten bacteriophages isolated from ten plaques obtained after the enrichment procedure of milk with *S. aureus* 916 were selected for further analysis.

### Bacteriophage genome sequencing and assembly

We extracted the genomes of the 10 isolates and proceeded to sequence them. Inspection of the de novo assemblies revealed one incomplete genome which was excluded from further analysis due to low sequencing coverage (6 ×). The nine assembled contigs showed the same length (43,129 bp) and GC content (35.11%). They were compared to each other with BLASTn to assess the coverage and identity percentages among them, which were both equal to 100%. Moreover, no SNPs could be detected, further highlighting their clonality. The subsequent analyses were conducted considering a unique clone among them, which was arbitrarily selected, and called phage Hesat. Its genome was assembled into a single contig, with a 114-fold coverage.

### Annotation and genetic characterization of phage Hesat

Phage Hesat shows the highest similarity with *S. aureus* phage phiJB (KT344895), with which it shares 73% and 98.87% of coverage and identity, respectively, indicating that Hesat belongs to the *Phietavirus* genus of the Azeredovirinae subfamily, which is not yet classified into a new taxonomic family of the Caudoviricetes. Phage Hesat can be considered a novel species, according to guidelines set out by Turner et al. (2021).

**Fig. 1:**
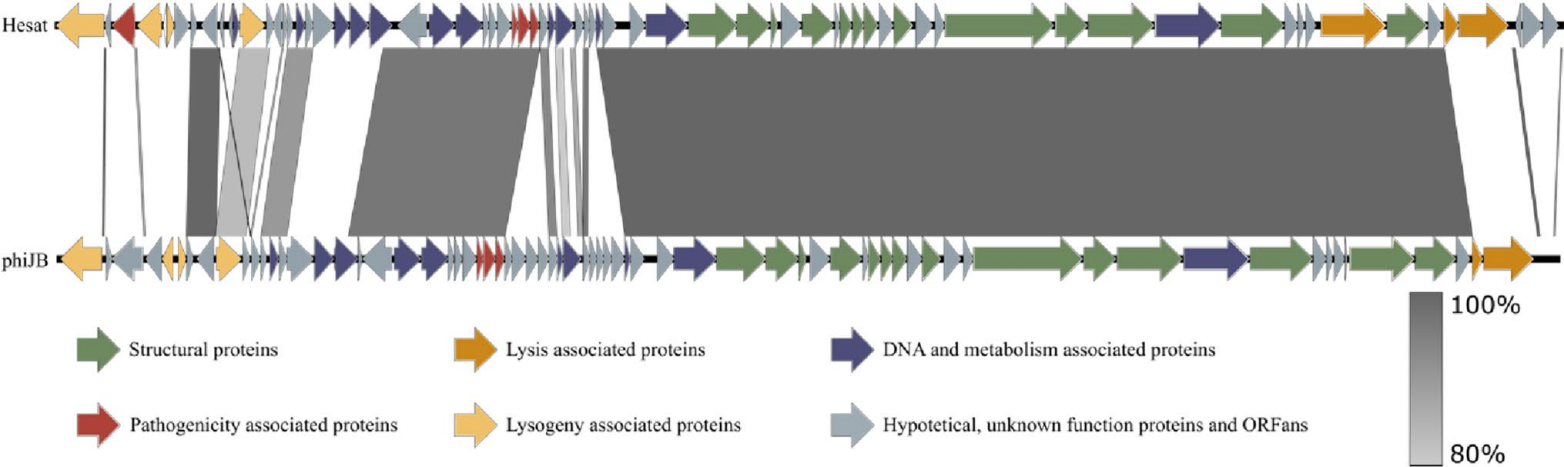
Genome organization of phage Hesat compared with phage phiJB. The arrows indicate annotated ORFs as follows: green arrows indicate ORFs associated with structural proteins, orange ORFs that encode for lysis-associated proteins, and yellow ORFs for lysogeny-associated proteins; purple ones DNA- and metabolism-associated proteins; red ORFs that encode for pathogenicity-associated proteins; in gray hypothetical, unknown function proteins and ORFans. The color intensity of the bands between two compared sequences indicates BLASTn percentage similarity

The phage genome (Fig. 1) was annotated with 75 open reading frames (ORFs), four of which are classified as ORFans, since they do not show homology compared to any other known genes. The annotated genes include 35 hypothetical proteins with unknown function. The other 36 ORFs were classified, according to the predicted functional proteins they encode, as follows: 12 of them encode DNA- and metabolism-associated proteins and 13 structural proteins and lysis-associated genes. In terms of lysogeny-associated genes, four are associated to a temperate lifestyle and a few predicted virulence factors have also been observed.

The DNA processing proteins include two nucleases and two DNA-binding proteins as well as a helix-turn-helix transcriptional regulator, a transcriptional activator, and a replication initiation protein.

As expected, the 13 structural genes are clustered in the second half of the phage sequence, and they encode minor and major capsid proteins, tail, and tail fiber proteins and two tail tape-measure proteins. Among the structural genes, an ORF coding for a connector protein was included. Being located at the capsid portal vertex, this connector protein is involved in bacteriophage tail attachment (Orlova et al. 2003).

An interesting feature within the phage Hesat genome is the encoded virulence factors. The first pathogenicity island (SaPln2) identified in the Hesat genome is 226 aa long and shares 100% sequence identity to the type II toxin-antitoxin system PemK/MazF family toxin, present in multiple species (Yamaguchi et al. 2011). A second relevant gene is *gp30*, coding for a DUF3113 protein, putatively involved in the derepression of the global repressor Stl, and whose binding might induce the expression of pathogenicity islands (Cervera-Alamar et al. 2018). A third gene coding for a virulence-associated factor is *SAV1978*, previously identified as a distinctive element of *S. aureus* prophages, but whose molecular function remains unknown (Bae et al. 2006). Finally, we annotated a gene coding for a Panton-Valentine leukocidin (PVL). PVLs are cytotoxins produced by S. *aureus*, often associated with MRSA strains (Vandenesch et al. 2003), and are responsible for leukocyte destruction and tissue necrosis (Genestier et al. 2005).

The temperate nature of phage Hesat was further confirmed by the presence of four genes coding for proteins involved in the lysogenic cycle, including two phage repressors and one phage antirepressor. They are responsible for the genetic switch between the lytic and lysogenic cycles during the infection (Heinrich et al. 1995). Moreover, *gp1* encodes a serine recombinase of the resolvase/invertase family, which generally mediates site-specific integration of phage DNA (Thorpe and Smith 1998).

The lysis cassette of phage Hesat includes two endolysins (*gp67* and *gp71*) and a phage holin (*gp70*). The former are peptidoglycan hydrolases that specifically cleave the amide bond between the lactyl group of the muramic acid and the α-amino group of l-alanine, causing disruption of the peptidoglycan structure (Foster 2004); the latter, the phage holin, is a small membrane protein, with two predicted transmembrane domains, which is necessary for host lysis (Wang et al. 2000).

No genes encoding proteins associated with acquired bacterial antibiotic resistance were predicted.

### Putative endolysin analysis

The two predicted endolysins were further characterized in order to better clarify their role with phage infection process and their different target molecules. Their structure was firstly determined through the prediction of functional domains and later of their tridimensional structure. *gp67* (Fig. S1a) was 624 aa long and it displayed at the N-terminus a cysteine, histidine-dependent amidohydrolase/peptidase domain (CHAP, residues 29–119) and a glucosamidase domain at the C-terminal (residues 485–613); in the *gp71* (Fig. S1b), 481 aa long, three domains were assessed. At the N-terminal a CHAP domain (residues 21–113), an amidase in the central region (residues 196–324) and a SH3 domain (residues 395–460) at the C-terminal. The CHAP domain is responsible of the cleavage of between D-alanine and the first glycine of the pentaglycine cross-bridge (Becker et al. 2009a); the glucosamidase hydrolyzes the N-acetylglucosaminyl β-1,4-N-acetylmuramine bond while the amidase cleaves between the sugar and the peptide moieties (Gutiérrez et al. 2018). Finally, the SH3_5 domain is involved in cell wall recognition and binding (Becker et al. 2009b).

### Phage Hesat appears to be a recombinant prophage of *S. aureus *strain 916

To assess whether phage Hesat was a prophage of the host strain *S. aureus* 916, the genome sequencing and analysis of the bacterial strain were performed. MLST analysis revealed that strain 916 belongs to the clonal complex CC1 (ST1), one of the five largest and most important *S. aureus* CCs. This complex is also known to contain various community-associated methicillin-resistant strains (Dabul and Camargo 2014). Moreover, strains belonging to CC1 have been isolated from both healthy/infected humans and animals ( Shepheard et al. 2013; McCarthy et al. 2012).The alignment between phage Hesat and its bacterial host genome (*S. aureus* strain 916) resulted in a coverage percentage of 91% and an identity of 100%, allowing to hypothesize that phage Hesat is a temperate phage integrated in its bacterial host genome as a prophage, although some rearrangements might have occurred during phage excision. When the reads from the bacterial strain sequencing were mapped against the phage genome, an average coverage of 40 × was observed throughout the phage genome length, besides a 3.7-kb region that was not covered (Fig. S2). After bacterial genome annotation, eight genes were identified in the region that do not match with the phage genome. Despite these eight genes not having the same sequences of those present in phage Hesat genome, it was interesting to notice that they are functionally close, since in both organisms, the annotated genes are related to the phage lysogenic cycle, indicating a potential recombination of the lysogeny decision cassette (Fig. S2).

The lack of the phage region within the bacterial genome was also verified through the PCR assay. Only in samples where phage DNA was included (lanes 2 and 3 in Fig. S3) the presence of the 405 bp fragment was observed, while the amplification did not occur neither by incubating the bacterial stock in the PCR reaction (lane 4) nor by adding the bacterial genome DNA samples (lanes 5 and 6). This result allowed to exclude the presence of the unmatching fragment within the bacterial strain.

By aligning the phage genome and the host, we determined the bacterial genome regions flanking the phage. On the left-hand side, a complex of iron-regulated surface determinant proteins was annotated; at the right-hand side, the ribosomal protein L32, associated with the large ribosomal subunit 50S, was detected.

To assess whether phage Hesat species was already circulating among further bacterial strains of both human and animal origins, a comparative analysis between its sequence and further predicted prophages was carried out as described at 2.6.

The genes submitted for BLAST analysis were selected taking into account their position along the phage genome and their functions: the integrase (*gp1*) was selected since primarily involved in lysogeny cycle and according to its position at the beginning of the phage sequence; the pathogenicity island SaPIn2 (*gp3*) and the PVL (*gp31*) were taken into account because of their epidemiologic role once integrated in the host; finally, the major capsid protein was included as structural gene, towards the right end of the sequence.

When the gene sequences were submitted to BLASTn, a different number of matching strains was obtained (242 for the integrase, 250 for the SaPIn2, 1375 for the PVL, 181 for the major capsid protein). Among them, only 16 strains, all belonging to *Staphylococcus aureus* species, shared all four genes at the same time. The PHASTER prediction, performed on these 16 strains, allowed to predict 49 putative prophages. From their alignment to Hesat sequence, only 11 out 49 shared with it at least a similarity of 70%, allowing to attribute them the same Hesat genus, but none belonged to the same species. As reported in the Table 2, the geographic origins of the bacterial strains were various, since they were isolated in four different continents (America, Africa, Asia, and Europe). In nine out of 11 cases, the isolation source was a human infection; in one case, the indicate source was “hospital,” while in another case, it was not specified.

**Table 2:** Predicted prophages from *Staphylococcus* strains annotated in NCBI databank and sharing at least the 70% of homology with phage Hesat

Predicted phage ID	Host strain	Integration region	Isolation source	Origin	GenBank ID	Similarity to Hesat (%)
P1_1	*Staphylococcus aureus* 59,731	1084786–1148445	Human infection	Tokyo, Japan	AP024738.1	80.81
P14_2	*Staphylococcus aureus* RM8376	1721349–1765440	n.a	n.a	NZ_CP064389.1	78.22
P9_2	*Staphylococcus aureus* FDAARGOS_766	749330–799917	Hospital	Lisbon, Portugal	NZ_CP041010.1	78.21
P3_3	*Staphylococcus aureus* P3.1	966008–1031553	Human blood infection	n.a	NZ_CP058312.1	78.05
P13_3	*Staphylococcus aureus* NP66	1678093–1739966	Human pus aspirate	Gauteng, South Africa	NZ_CP041037.1	76.98
P10_4	*Staphylococcus aureus* Gv51	1165154–1208,620	Human pneumonia	Teresina, Brazil	NZ_CP012015.1	75.88
P16_5	*Staphylococcus aureus* Gv69	1390053–1433415	Human patient with hospital-associated wound infection	Teresina, Brazil	CP009681.1	75.78
P11_5	*Staphylococcus aureus* HC1340	1214242–1257359	Patient associated to home care assistance	Rio de Janeiro, Brazil	NZ_CP012011.1	74.81
P12_5	*Staphylococcus aureus* MN8	2058065–2116431	Human patient with toxic shock syndrome	USA	NZ_CP091875.1	72.55
P6_4	*Staphylococcus aureus* ER02878.3	1820206–1866974	Human blood infection	New York City, USA	CP030713.1	71.91
P8_3	*Staphylococcus aureus* ER11063.3	1158504–1205272	Human blood infection	New York City, USA	CP051914.1	71.90

**Fig. 2:**
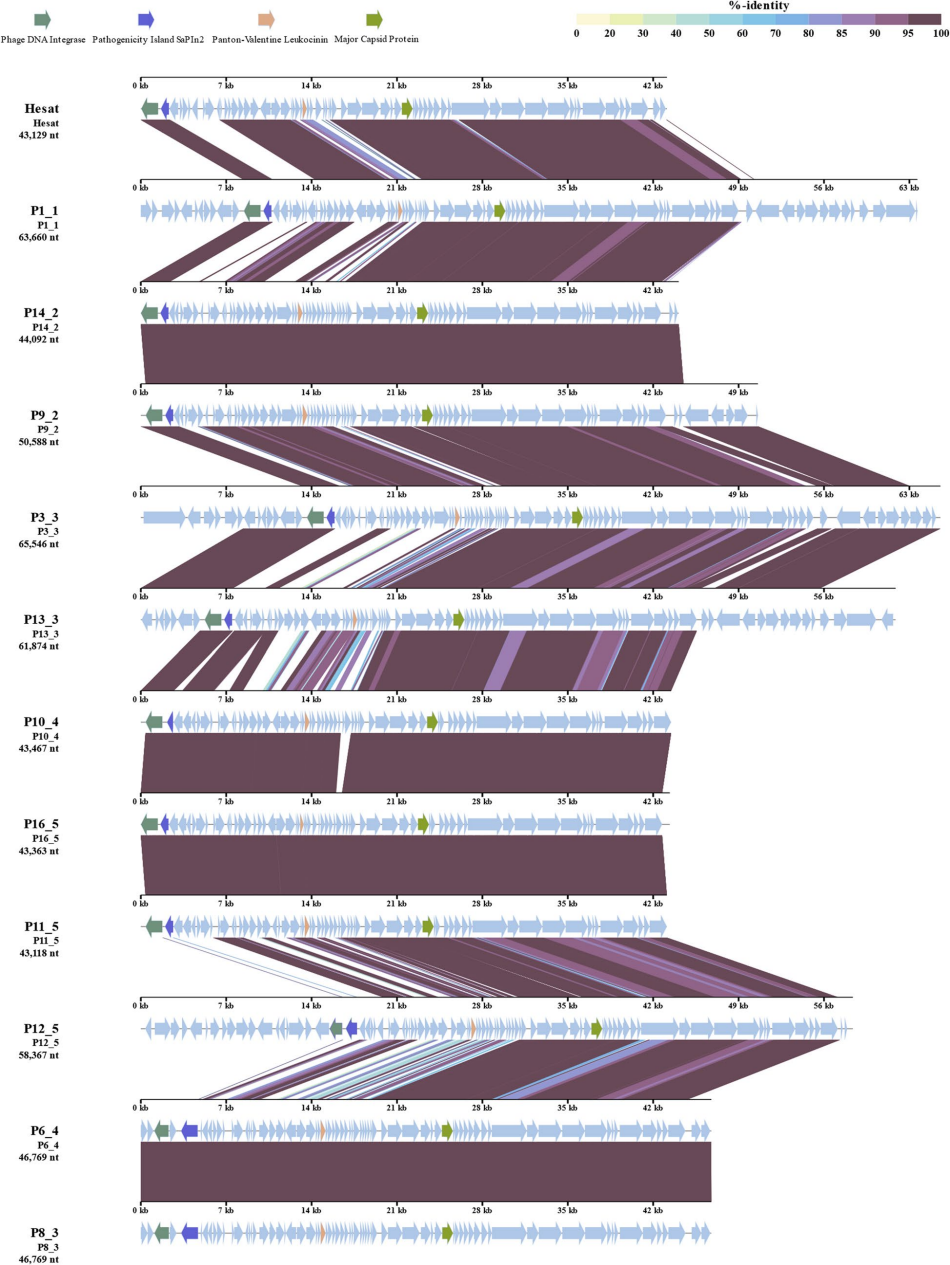
Genomic similarity of Hesat to 11 prophages found in different *Staphylococcus aureus* strains. The alignment was generated using VipTree (Nishimura et al. 2017). The arrows represent the integrase (dark green), the pathogenicity island SaPIn2 (purple), the Panton-Valentine leukocidin (orange), and the major capsid protein (light green). Genome identity, indicated in iridescent color scale, represents the sequence similarity, based on a tBLASTx alignment

Finally, the prophage genome alignment (Fig. 2) indicated that there is more variability in the first half of the sequences, where most of lysogeny-associated proteins, toxins, and metabolism-associated proteins are present, while the second half of the genomes, carrying the structural proteins, is more conserved.

### Bacteriophage host range

Among the 23 isolates of human origin (including ATCC reference strains), 21 spa types were detected. Spa-type t127 was the only one shared between human and dairy isolates (Table 1). The host range of phage Hesat was assessed performing a spot assay with three different titers (10^8^ PFU/ml as high titer, 10^6^ PFU/ml as medium titer, and 10^4^ PFU/ml as low titer) against all the *S. aureus* isolates included in the study. We also tested it against nine coagulase-negative staphylococcal strains belonging to different species (*S. epidermidis*, *S. simulans*, S. *chromogenes*, *S. equorum*, *S*. *arlettae*, *S*. *xylosus*, *S*. *auricularis*, *S*. *jettensis*, and *S*. *muscae*).

As for the isolates of dairy origin, four out of seven were lysed by phage Hesat, both at a titer of 10^8^ and 10^6^ PFU/ml. Furthermore, isolates 30 and 916 were also lysed at the lowest phage concentration (Table 3). By contrast, isolates 18, 153, and 224 could not be lysed by phage Hesat. Concerning the coagulase-negative staphylococcal species, *S. muscae* isolate was the only strain to be lysed by phage Hesat, when tested at the highest titer.

**Table 3:**
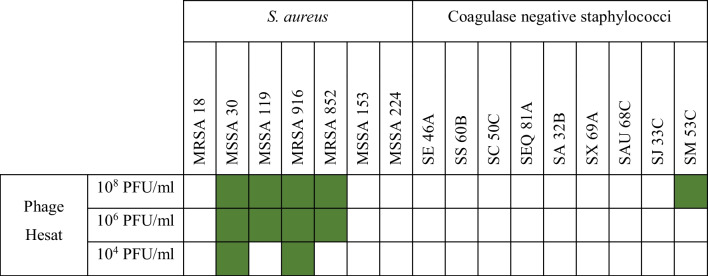
Host spectrum of phage Hesat versus staphylococcal strains of dairy origin

SE *S. epidermidis*, SS *S. simulans*, SC *S. chromogenes*, SEQ *S. equorum*, SA* S. arlettae*, SX* S. xylosus*, SAU *S. auricularis*, SJ *S. jettensis*, SM* S. muscae*

Eighteen out of 21 strains display sensitivity to phage Hesat lytic activity, when spotted at the highest titer (Table 4). Five strains were also susceptible to the medium phage titer of 10^6^ PFU/ml, and this was observed at least for one strain coming from each country of the dataset (the Italian SA VT, the German 9, the Belgian V-190821–119, and the Swiss AOMu100, and AOC42). Moreover, a lytic effect of phage Hesat was shown when tested at the lowest titer against two *S. aureus* strains (9 and V-190821–119).

**Table 4:**
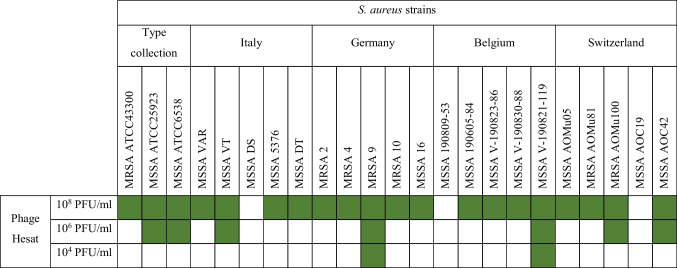
Host spectrum of phage Hesat versus *S. aureus* strains of human origin

## Discussion

Phage therapy is regaining interest for the treatment of bacterial infections caused by drug-resistant pathogens or biofilm-associated microorganisms (Strathdee et al. 2023), including *S. aureus*. Due to the extreme selectivity of phages (at strain level), finding new phages expressing novel proteins is crucial for the development of phage therapy.

As most of the published *S. aureus* temperate phages, Hesat is a siphovirus. In particular, it is a novel species within the *Phietavirus* genus. Viruses of this genus present an icosahedral head of about 50 nm in diameter and a non-contractile tail of approximately 175 nm. Besides phage Hesat, 30 other annotated phage species belong to the *Phietavirus* (ICTV MSL38 v2). Despite the structural closeness, *Phietavirus* phages differ in their genomes as well as in the effects on *S. aureus* strain pathogenicity. Some of them carry genes encoding virulence factors (Goerke et al. 2009; Yamaguchi et al. 2011); others were identified as strongly involved in horizontal transfer of pathogenicity islands (SaPIs) (Christie et al. 2010). Phages of a third group, carrying neither antibiotic-resistance genes nor known toxin genes, are considered putative candidates for phage therapy (Matsuzaki et al. 2003).

When the genome sequence of phage Hesat was aligned to further prophages predicted from previously annotated bacterial strains, we confirmed that no phages belonging to the same Hesat species were present, but 11 predicted prophages shared its genus. This could be due to a high mutation frequency in lysogeny-associated genes, toxins, and metabolism-associated genes, which are more distributed in the first half of *Phietavirus* phage sequences. Conversely, genomic variability was lower in the regions carrying genes coding for structural proteins.

Although we cannot exclude that Hesat is a temperate phage commonly circulating in dairy environment and in raw milk samples collected in Tuscany region, we propose that it derives from the prophage of *S. aureus* 916. Indeed, a perfect sequence identity with the prophage was observed in the 91% of the coverage of the Hesat’s 43,129 bp genome. It is possible that recombination events with a different prophage (carried by extrachromosomal DNA elements present in milk samples) might have occurred during its excision, generating a new phage with a 3.7-kb different region.

*S. aureus* 916 was isolated from bulk tank milk in the South of Italy and it belonged to spa-type t127. Spa-type t127 was the most frequently detected among the *S. aureus* isolates included in this study. Spa-type t127 isolates were reported among the most prevalent clones, both methicillin-susceptible *S. aureus* (MSSA) and MRSA, from human invasive infections in Europe (Grundmann et al. 2010; Cuny et al. 2015). Spa-type t127 generally includes human community-associated MRSA and Panton-Valentine leukocidin (PVL)–positive or PVL-negative strains. It was also isolated from animals, such as cattle (Juhász-Kaszanyitzky et al. 2007; Huber et al. 2010; Pilla et al. 2012; Hummerjohann et al. 2014) and pigs (Hasman et al. 2010; Franco et al. 2011). 

Phage DNA may integrate into the host genome either in non-coding sequences or within coding sequences, interrupting host genes. Phage Hesat integrates in a non-coding sequence, flanked by a cluster of iron-regulated surface proteins and by the 50S ribosomal protein L32. In a previous study (Keary et al. 2014), the latter region was reported as one of five high-frequency integration loci (two non-coding sequences and three host genes), suggesting a high level of integration specificity.

We also observed that mitomycin C was not effective in inducing the excision of prophages from *S. aureus* 916. However, incubating the bacterial strain in raw milk at the conditions proposed by García et al. (2009) increases the recovery of phages particles. This concurs with the observation by Humphrey et al. (2021), who suggested that milk represents a more natural model for studying phage- or PICI-mediated plasmid transfer. Indeed, it is known that bulk tank milk is characterized by the presence of psychrotrophic bacteria and subinhibitory concentrations of antimicrobial molecules (Decimo et al. 2014). This condition may promote prophage induction (Humphrey et al. 2021), resulting in the higher percentages of milk samples testing positive for phage presence.

The host range of phage Hesat was rather broad, with the phage showing lysis on 24 out of 30 tested *S. aureus* isolates. The proportion of isolates which were not lysed was higher when considering those of dairy origin (3/7), while among isolates of human origin, the number of non-lysed isolates was lower (3/23). However, it is not known whether the inability to form plaques observed was the result of phage integration in *S. aureus* strain genomes. This relatively wide host range suggests a potential high diffusion of phage Hesat in different environments, contributing to the spreading of pathogenicity traits among *S. aureus* strains of human and animal origin. It was interesting to observe that among resistant isolates of dairy origin, *S. aureus* 18 belonged to the same spa type (t127) as *S. aureus* 916. Furthermore, when tested at highest titer (10^8^ PFU/ml), Hesat phage was also able to lyse *S. muscae* 53C. Although reports of interspecies staphylococcal phage are rather uncommon, it is possible that *S. mus*cae 53C and *S. aureus* 916 strains share some properties in the wall teichoic acid cell surface receptors resulting in the attachment of Hesat also on this coagulase-negative strain causing its lysis, probably from without.

Although strictly lytic phages have been preferred for phage therapy, mostly to avoid potential transduction, Hesat might also be explored for the delivery of protein encoding genes targeting bacterial antibiotic resistance or virulence factors (Monteiro et al. 2019). Moreover, it is also possible to engineer the genome of temperate phages to eliminate lysogeny determinants (Tinoco et al. 2016; Dedrick et al. 2019). By doing so, temperate phages become lytic and can be used as any virulent phage for therapeutic treatment.

Finally, temperate phages are also a good source of endolysins which can be used as enzybiotics for the treatment of antibiotic-resistant strains, especially versus gram-positive bacteria (Rodríguez-Rubio et al. 2016). Moreover, endolysins are also considered good bioreceptors for the detection of bacteria as they have a high affinity and specificity towards the ligands on the gram-positive cell wall (Sumrall et al. 2020). Here, we found novel putative endolysis which might be further explored for its application as either antimicrobials or biosensors. In conclusion, a novel phage targeting both animal and human *S. aureus* strains has been found and further studies based on genome editing will be carried out.

## Supplementary Information

Below is the link to the electronic supplementary material.

Supplementary file1 (PDF 305 KB)

## Data Availability

The datasets generated during and/or analyzed during the current study are available from the corresponding author on reasonable request. All bacterial and bacteriophage samples are also available upon request. The complete nucleotide sequence of the phage Hesat genome was deposited to NCBI GenBank and is available under accession no. OP947204. The bacterial strain *Staphylococcus aureus* 916 was deposited in BCCM/LMG Bacteria Collection as LMG 33221, and its genome is available in the NCBI GenBank under accession no. JAWJEE000000000.
