# Supplementary material for: Isolation and characterization of novel Staphylococcus aureus bacteriophage Hesat from dairy origin
Source: Appl Microbiol Biotechnol. 2024 Apr 15;108:299. doi: 10.1007/s00253-024-13129-y (PMC11018700; doi:10.1007/s00253-024-13129-y)
Supplement: Supplementary file 1 [file 253_2024_13129_MOESM1_ESM.pdf]

Applied Microbiology and Biotecnology

Isolation and characterization of novel *Staphylococcus aureus* bacteriophage Hesat from dairy origin

Barbara Turchi[a,#,*], Claudia Campobasso[b, c,#], Arianna Nardinocchi[b], Jeroen Wagemans[c], Beatrice Torracca[a], Cédric Lood[c,d], Graziano Di Giuseppe[b], Paola Nieri[e], Fabrizio Bertelloni[a], Luca Turini[a], Valeria Ruffo[a], Rob Lavigne[c], Mariagrazia Di Luca[b]

[a]Department of Veterinary Science, University of Pisa, Viale Delle Piagge 2, 56124 Pisa, Italy

[b]Department of Biology, University of Pisa, Via San Zeno 37, 56127 Pisa, Italy

[c]Department of Biosystems, KU Leuven, Kasteelpark Arenberg 21, box 2462, 3001 Leuven, Belgium

[d]Department of Microbial and Molecular Systems, Centre for Microbial and Plant Genetics, KU Leuven, Kasteelpark Arenberg 20, box 2460, 3001 Leuven, Belgium[e]

Department of Pharmacy, University of Pisa, Via Bonanno Pisano 6, 56126 Pisa, Italy

*Corresponding author: mariagrazia.diluca@unipi.it, Department of Biology, Via San Zeno 37, 56127 (Pisa), Italy, phone: +390502213697

# Equally contributed

Supplementary figures

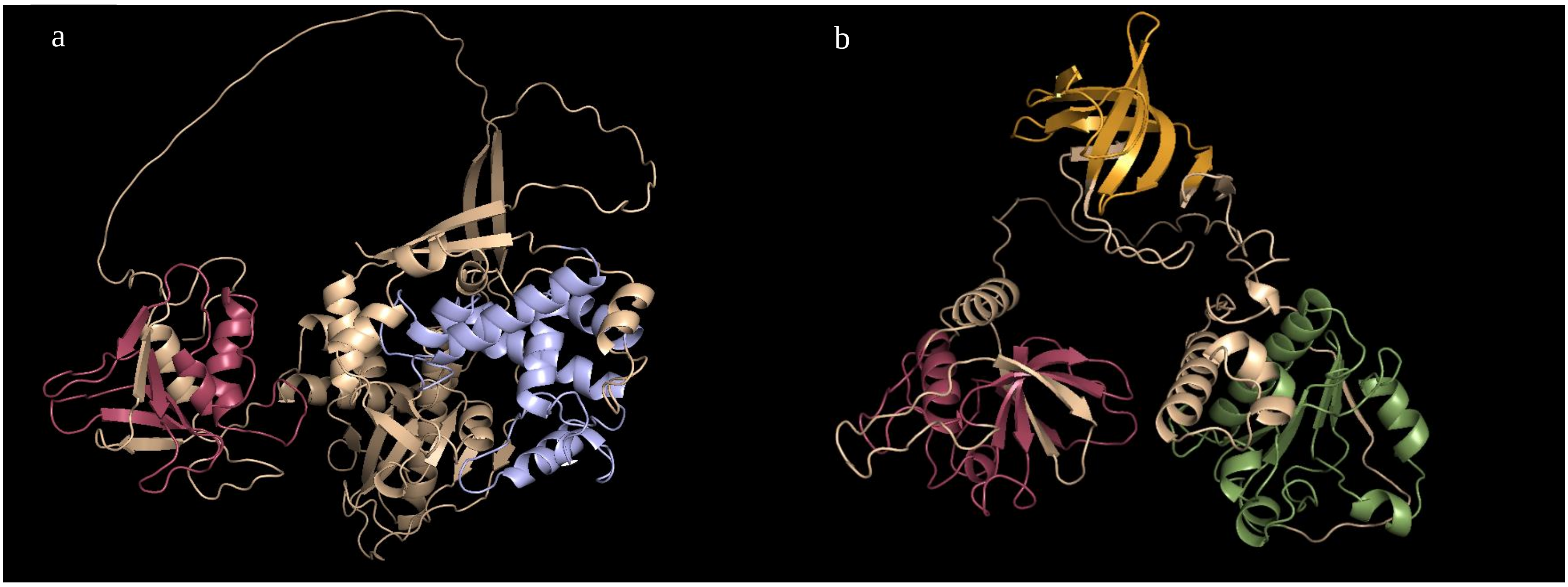

Fig. S1 Tridimentional structure of the endolysins a) *gp67* and b) *gp71*, predicted by ColabFold. The backbones are displayed in beige while the functional domains are highlighted as follows: in pink the CHAP domains, in purple the glucosamidase, in green the amidase and in yellow the SH3_5 domain.

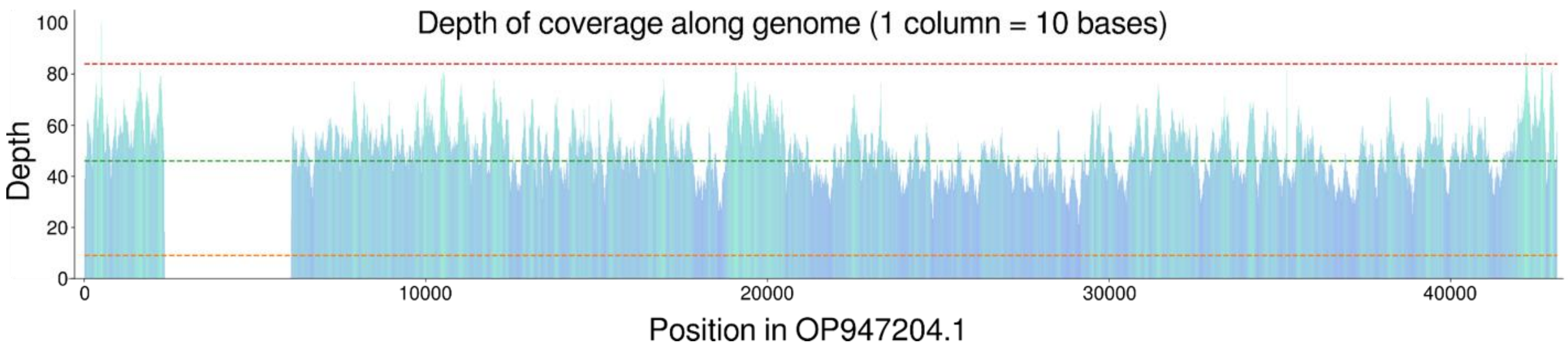

Fig. S2 Mapping of the reads from the *S. aureus* 916 strain sequencing against the phage Hesat genome reveals a coverage of about 40× all along the phage genome, besides the 3.7 kbp recombined region that is uniquely found in the temperate phage

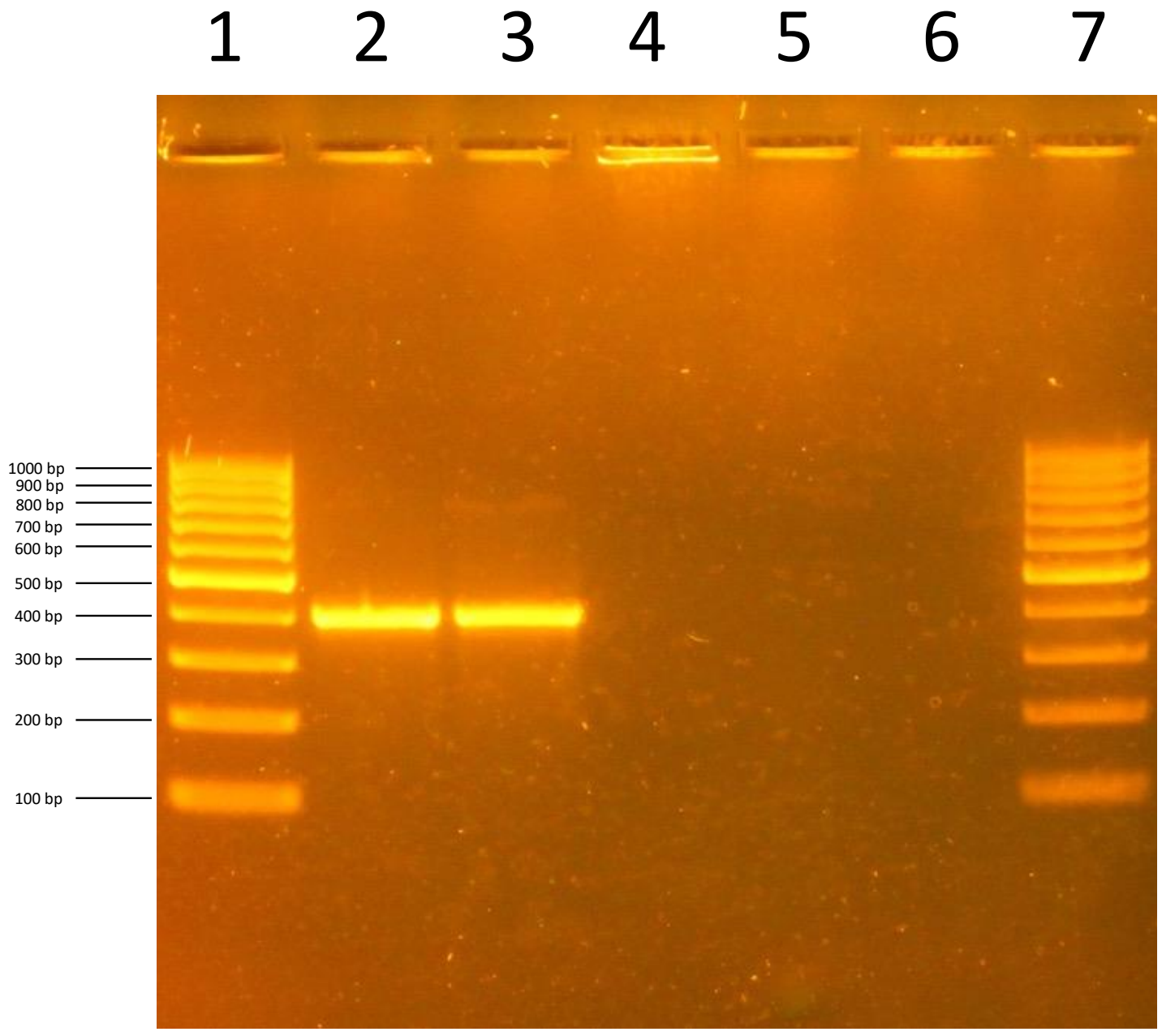

Fig. S3 Agarose gel electrophoresis (2% agarose) of the PCR product of the 405 bp fragment amplification: lane 2 and 3, two samples of phage Hesat lysate, lane 4 *S. aureus* 916 strain glycerol stock, lanes 5 and 6 two samples of the bacterial genome DNA. In the lanes 1 and 7 the GeneRuler 1kb DNA ladder
